# Simulation of advanced cataract surgery – validation of a newly developed test

**DOI:** 10.1111/aos.14439

**Published:** 2020-04-18

**Authors:** Mads Forslund Jacobsen, Lars Konge, Morten la Cour, Lars Holm, Hadi Kjærbo, Birgitte Moldow, George M. Saleh, Ann Sofia Skou Thomsen

**Affiliations:** ^1^ Department of Ophthalmology Rigshospitalet – Glostrup Copenhagen Denmark; ^2^ Copenhagen Academy for Medical Education and Simulation Centre for HR Capital Region of Denmark Copenhagen Denmark; ^3^ NIHR Biomedical Research Centre at Moorfields Eye Hospital and UCL Institute of Ophthalmology London UK

**Keywords:** cataract surgery, Eyesi, medical education, simulation, virtual reality

## Abstract

**Purpose:**

To develop and investigate an Eyesi simulator‐based test for the more experienced cataract surgeon for evidence of validity.

**Methods:**

The study was a prospective interventional cohort study and carried out at the Copenhagen Academy for Medical Education and Simulation. The Eyesi Simulator was used for the test which was developed by three expert cataract surgeons. Ten cataract surgeons (>250 surgeries performed) and ten ophthalmic residents performed two repetitions of the test. The test consisted of four modules: Iris Expansion Ring insertion – level 1, Iris Expansion Ring extraction – level 2, Capsulorhexis – level 3 and Anterior Vitrectomy – level 6.

**Results:**

Internal consistency reliability showed Cronbach’s alpha of 0.63. Test–retest reliabilities were significant for Iris Expansion Ring extraction – level 2 (p = 0.012) and Capsulorhexis – level 3 (p = 0.018). Differences between the two groups were only significant in both repetitions for the Iris Expansion Ring extraction – level 2 (p < 0.001 and p = 0.041, respectively). Furthermore, we found a statistically significant difference between the mean module scores for novices and the more experienced surgeons for Iris Expansion Ring insertion – level 1 (p = 0.021) and Capsulorhexis – level 3 (p = 0.019) in the first repetition.

**Conclusion:**

The investigated modules show evidence of validity within several aspects of Messick’s framework. However, the evidence is not strong enough to apply the test for certification purposes of cataract surgeons, but the modules may still be relevant in the training of advanced cataract surgical procedures.

## Introduction

Cataract surgery is the most common surgical procedure performed in the European union (Eurostat 2019). It is a microsurgical procedure that requires mastery of complex visuospatial techniques (Staropoli et al. 2018). Traditionally, cataract surgical abilities are developed through many years of experience in the operating theatre. However, there is an increased risk of complications during the first part of the surgeon’s learning curve (Randleman et al. 2007; Sparrow et al. 2011; Haripriya et al. 2012). Consequently, a safe training environment for surgeons to hone their skills and develop their abilities without risk for the patients is needed.

Previously, this safe training environment was supplied by different models such as inanimate models and porcine eyes. However, despite being accessible and offering moderate to high levels of physical resemblance and functional task alignment, there are certain disadvantages such as infection risks (including methicillin‐resistant staphylococcus aureus) and specific dissimilarities in the biomechanical properties compared with the human eye (Thomsen 2017). With the introduction of virtual reality (VR)‐based simulation, the possibility of constant feedback and part‐task training emerged (Thomsen 2017). Since the early 2000’s, the Eyesi Simulator (vr_magic_, GmbH, Mannheim, Germany) has enabled surgeons to train procedural and abstract tasks related to cataract surgery, while providing continuous feedback on performance (Khalifa et al. 2006). In the training of cataract surgical skills, the Eyesi Simulator has shown to improve surgical performance in the operating room and reduce complication rates (Thomsen et al. 2017; Ferris et al. 2020). It has become an integral part of cataract surgical education programs in many ophthalmology departments worldwide. However, structured training on the Eyesi Simulator has previously only shown to have an effect on novice surgeons (Thomsen et al. 2017).

For the more experienced cataract surgeon, previous studies have not been able to detect a clinically significant effect from simulator training on operating room performance (Thomsen et al. 2017). This finding may have been influenced by the fact that the simulator modules investigated in previous studies all focused on basic cataract surgical tasks, as no Eyesi Simulator modules designed for advanced cataract surgical tasks and procedures existed. However, it is highly relevant for the intermediate and experienced cataract surgeon to be able to practice the handling of surgical complications and difficult clinical cases in a safe environment, before they are gradually exposed to them in the operating room.

New modules designed for the training of advanced cataract surgical procedures have now been included in the newest software version of the Eyesi Simulator. These modules are designed for the more experienced cataract surgeons who wish to train the handling of surgical complications and more difficult clinical cases. The modules focus on procedures such as implantation and explantation of an Iris Expansion Ring, performing a capsulorhexis on a white cataract and handling of posterior capsular rupture by Anterior Vitrectomy. However, because of the novelty of these modules, no studies have investigated them for evidence of validity, which is needed in order to ensure that they truly measure what they claim to measure: advanced cataract surgical skill.

Our aim with the current study was to explore validity evidence for the newly developed advanced cataract surgical modules on the Eyesi simulator, using Messick’s contemporary validity framework.

## Methods

### Study design

The study was designed as a prospective interventional study. The study was conducted at the Copenhagen Academy for Medical Education and Simulation (CAMES). The Ethics committee of the Capital Region of Denmark ruled that approval was not required for this study (protocol no. H‐18052332). The study adheres to the tenets of the Declaration of Helsinki.

### Participants

We aimed to include 20 participants in the study. No sample size calculation was performed for this study due to the novelty of the investigated modules. Instead, we based our study size on a previous validity study investigating basic cataract surgical modules on the Eyesi Simulator (Thomsen et al. 2015) and the availability of eligible study participants. The participants were ophthalmology residents and cataract surgeons (>250 surgeries performed). Three groups of participants were defined: (1) Novices; ophthalmology residents without any cataract surgical experience, (2) Experienced; cataract surgeons with 250–2000 cataract procedures performed and (3) Experts; cataract surgeons with >2000 cataract procedures performed. Surgeons and novices who had trained for more than 2 hr on the Eyesi simulator in the past six months were not eligible to participate in the study due to the risk of bias. All participants gave oral and written consent before they were included in the study. Participants completed a questionnaire regarding age, dexterity, stereopsis and surgical experience. Stereo acuity was measured using the TNO test (Laméris Ootech BV, 19th edition).

### Data collection

The cataract interface on the Eyesi Simulator (vr_magic_, software version 3.4.2) was used. Three expert cataract surgeons (BM, HK and LM) selected modules and difficulty levels corresponding to relevant learning objectives for a cataract surgeon with >100 operations performed. See Table 1 for an overview of the included modules and the chosen difficulty level of each task.

**Table 1:** Overview of included modules.

Module no.	Task name	Task description	Module Levels	Chosen level	Points
1	Iris Expansion Ring	Insert a Malyugin ring onto the iris	2	1	0–100
2	Iris Expansion Ring	Extract a Malyugin ring from the iris	2	2	0–100
3	Capsulorhexis	Perform a continuous curvilinear capsulorrhexis on a white cataract	4	3	0–100
4	Ant. Vitrectomy	Perform an anterior vitrectomy after rupture of posterior capsule during irrigation and aspiration then finish irrigation and aspiration procedure.	7	6	0–100

The four modules each have a maximum score of 100 points. This score is based upon performance within each of five different domains: target achievement, efficiency, tissue treatment, instrument handling and microscope handling.

All participants received a 10‐min basic introduction to the simulator followed by a familiarization (warm up) session consisting of one repetition of the program. Thereafter, the participants completed two final repetitions of the program. During the program, one author (MFJ) gave instructions and explained all procedural and performance goals to the participants. Participants were instructed to follow instructions as guided by the simulator. This was done to minimize factors other than technical ability that could influence performance. The data collected were the metrics from the two final repetitions of the program. Messick’s framework of validity was used to interpret the results of this study. Messick’s framework is a modern framework considered the gold standard when evaluating validity evidence. It consists of five different sources of validity evidence: content, response process, internal structure (i.e. reliability), relations with other variables and consequences of the assessment (Downing & Yudkowsky 2009; American Educational Research Association APA 2014; Cook et al. 2014).

### Statistical analysis

ibm spss statistics 22.0 (SPSS Inc., Chicago, IL, USA) was used for statistical analysis. Internal consistency reliability between modules was determined by calculating Cronbach’s alpha. Cronbach’s alpha is a measure of how closely related a set of items are as a group, and it is a tool to helps us understand how well a test measures what it should (e.g. advanced cataract surgical skill). Cronbach’s alpha ranges between 0 and 1, with higher values indicating greater internal consistency reliability. The Pearson correlation coefficient was used to investigate the test–retest reliability between the two test repetitions. One‐way anova was performed to compare the mean test scores for novice, experienced and expert participants. A discriminative ability on a 5% level was considered statistically significant.

## Results

Twenty participants were included in the study. All 20 participants completed the study and were included in the final data analysis. Table 2 shows the descriptive data for the three groups of participants. There were no significant differences in dexterity or stereopsis between the groups.

**Table 2:** Group characteristics.

	Novices	Experienced	Expert
*n* (20)	10	5	5
Gender (%‐female)	70%	20%	60%
Age median (range)	32 (30; 38)	43 (38; 44)	43 (38; 54)
Dexterity (%‐right handed)	90%	100%	80%
TNO mean (range)	60 (60; 60)	60 (60; 60)	60 (60; 60)
Total no. of phacoemulsifications performed, median (range)	0 (0; 0)	1000 (274; 1800)	8200 (3000; 15 000)
Total no. of Malyugin ring insertions performed, median (range)	0 (0; 0)	15 (0; 100)	80 (20; 200)
Total no. of capsulorhexis on white cataracts performed, median (range)	0 (0; 0)	2 (2; 10)	285 (10; 1000)
Total no. of ant. vitrectomies performed, median (range)	0 (0; 0)	10 (1; 20)	56 (5; 100)
Total no. of intravitreal injections performed, median (range)	400 (0; 2000)	500 (0; 2000)	100 (0; 500)
Total no. of glaucoma related operations performed, median (range)	0 (0; 0)	0 (0; 0)	0 (20; 500)
Total no. of pterygium related operations performed, median (range)	0 (0; 0)	0 (4; 10)	50 (0; 500)
Total no. of vitreoretinal operations performed, median (range)	0 (0; 0)	0 (0; 0)	0 (0; 100)

An overview of mean module scores for each test repetition for novices, experienced and expert cataract surgeons is available in Table 3.

**Table 3:** Overview of module score for novices, experienced and expert cataract surgeons.

Module	Novice mean score Test 1 (SD)	Novice mean score Test 2 (SD)	Experienced mean score Test 1 (SD)	Experienced mean score Test 2 (SD)	Expert mean score Test 1 (SD)	Expert mean score Test 2 (SD)	p‐Value (test 1)	p‐Value (test 2)
Iris Expansion Ring insertion – level 1	19 (25)	21 (37)	7 (12)	55 (31)	57 (39)	37 (40)	**0.021**	0.227
Iris Expansion Ring extraction – level 2	9 (20)	15 (23)	77 (15)	65 (38)	51 (41)	45 (49)	**<0.001**	**0.041**
Capsulorhexis – level 3	24 (34)	41 (31)	72 (12)	65 (37)	56 (32)	75 (12)	**0.019**	0.132
Anterior Vitrectomy – level 6	37 (33)	64 (45)	75 (22)	55 (50)	39 (40)	67 (38)	0.120	0.903

Bold values indicate statistically significant of p < 0.05 level.

The internal consistency of the modules showed a relatively low Cronbach’s alpha of 0.63. There was a low but significant test–retest reliability between test repetition one and two for Iris Expansion Ring extraction – level 2 with a Pearson correlation of 0.55 (p = 0.012) and Capsulorhexis – level 3 with a Pearson correlation of 0.52 (p = 0.018). The remaining modules did not have significant test–retest reliability.

There were statistically significant differences between the mean module score for novices and the more experienced and expert surgeons for Iris Expansion Ring insertion – level 1 (p = 0.021), Iris Expansion Ring extraction – level 2 (p < 0.001) and Capsulorhexis – level 3 (p = 0.019), for the first repetition of the test. Only the Iris Expansion Ring extraction – level 2 showed a statistically significant difference between mean score for novices and the more experienced and expert surgeons for the second repetition of the test (p = 0.041). Post hoc analysis showed there was no statistically significant difference in the mean test scores between the experienced and expert cataract surgeons.

One module, the Anterior Vitrectomy – level 6 showed no ability to discriminate between novice, experienced and experts in either test repetition.

## Discussion

In the current study, we have demonstrated that it is possible to design a simulation‐based test for advanced cataract modules on the Eyesi Simulator. The results of our study suggest the presented test has a relatively low inter‐module and test–retest reliability with Cronbach’s alpha of 0.63 and a significant test–retest reliability for two out of four modules (Expansion Ring extraction – level 2 and Capsulorhexis – level 3). Only one module demonstrated discriminative ability between participants (Iris Expansion Ring extraction – level 2). To our knowledge, this is the first study to investigate advanced cataract surgical Eyesi modules.

Messick’s framework of validity consists of five sources of validity. The five sources of validity are content, response process, internal structure, relations to other variables and consequences of the assessment (Ghaderi et al. 2015).

In order to ensure content validity, we based the program on pilot testing and discussions with three expert cataract surgeons (BM, HK and LM). These three surgeons tested all available Eyesi modules and chose the advanced cataract surgical modules and difficulty levels with relevance for an independent cataract surgeon with >100 operations performed. This process of selecting relevant modules based on input from participants familiar with the procedure of interest is similar to previous validity studies in other surgical fields (Savran et al. 2015).

In this study, the response process consisted of measures to minimize the risk of bias during the data collection. This was done by using standardized written instructions supervised by a single instructor (MFJ) throughout the entire data collection and by relying solely on the simulator’s automated and objective metrics.

The internal structure refers to the exploration of the reliability of scores that seek to measure the same construct, for example skill, knowledge etc., and can be statistically evaluated using Cronbach’s alpha or other reliability indices (Bloch & Norman 2012). The reliability analysis provides insight regarding the reproducibility of test scores and gives us an understanding of the applicability of a test for different levels of assessment (i.e. formative assessment for feedback, summative assessment for certification etc.) (Downing 2004). We investigated the internal consistency reliability of the modules with discriminative ability using Cronbach’s alpha and found a significant but relatively low level of reliability between these items (Cronbach’s alpha 0.63). Furthermore, we found a significant test–retest correlation only for Iris Expansion Ring extraction – level 2 and Capsulorhexis – level 3 (Pearson’s correlation 0.55, p = 0.012 and 0.52, p = 0.018, respectively). For formative assessment purposes (i.e. feedback), Cronbach’s alpha of 0.70–0.79 is appropriate. However, for summative assessment purposes (i.e. certification), at least 0.80 is needed (Downing 2004). Consequentially, the program is not suited for high stakes assessment purposes but may still be relevant for training of advanced cataract surgical procedures. Other studies investigating validity evidence of the more basic cataract surgical modules have found an inter‐module reliability coefficient of 0.76 when investigating seven basic cataract surgical modules (Thomsen et al. 2015). Administering our test to medical students instead of ophthalmic residents would improve the reliability as the difference between medical students and experienced and expert cataract surgeons (i.e. ‘the signal’) is more pronounced (Norman 2008). However, this way of improving reliability (i.e. ‘the signal/noise ratio’) is not recommended as our target audience is the more experienced cataract surgeon, and therefore, a comparison with medical students would not be relevant.

A possible explanation of low reliability could come from participants receiving feedback on performance in between test sessions and thereby improving their performance through a testing effect. To minimize this, one instructor (MFJ) supervised the data collection using written instructions only and refrained from giving feedback. Another possible explanation could come from the familiarization effect. This effect relates to the fact that study participants may have different rates of familiarizing themselves with the Eyesi simulator and that familiarity with the equipment will influence test scores (Thomsen 2017). Therefore, it is important to include an introductory and warm‐up period before commencing the test, as was done in our study. The warm‐up period, however, must be of appropriate length; if it is too short, the participants will still be familiarizing themselves with the equipment during the test. However, if the warm‐up period is too long, the performance difference between novices and experienced surgeons will be significantly reduced. Given our results, it is possible that this warm‐up period may not have been long enough as results suggest that participants to some extent may still be familiarizing themselves with the equipment during the test repetitions.

The relation to other variables refers to the relationship between test scores and external or independent measures (Borgersen et al. 2018). These measures could include proficiency level and expertise. In the current study, this was examined using one‐way anova. The analysis showed that for the Iris Expansion Ring insertion – level 1, there was a significant difference in mean test scores between novice and experienced and expert participants for the first repetition of the test, but not the second repetition. Furthermore, our results showed that for the Iris Expansion Ring extraction – level 2, there was a significant difference between mean scores for novices and the experienced and expert participants for both repetitions of the test. For the Capsulorhexis – level 3, there was a significant difference in mean test score for the different groups of participants for the first repetition of the test. However, the novices’ test scores for the Capsulorhexis – level 3 almost doubled in the second repetition of the test, and therefore, there was an insignificant difference between mean test scores across groups. This significant improvement in mean module score from first to second repetition for novices was applicable for all four tested modules, reflecting the steep learning curve that novice participants likely undergo from first to second repetition. Meanwhile, experienced and expert participants did not consistently improve from first to second repetition across all four modules. The difference in performance and subsequent test scores for experienced and expert participants were not distinct enough to be statistically significant. This is to be expected as the surgical performance improves drastically in the first few hundred cases without reaching a plateau with increased surgical experience (Randleman et al. 2007). Consequentially, the difference in surgical performance between experienced and expert surgeons is too subtle to be statistically significant. The Anterior Vitrectomy level 6 did not show any ability to discriminate between novice, experienced and expert surgeons in any repetition. This may be due to the fact that performing an anterior vitrectomy following a posterior capsular rupture is a complex task, which can be approached in many different ways. The Eyesi simulator encourages a specific approach to this task and punishes alternative approaches. This may have an effect on the performance of the more experienced cataract surgeons, as they may be accustomed to one specific surgical approach and will struggle to readjust due to sheer force of habit. The novices are not inhibited by preferences and force of habit and therefore do not struggle to readjust their performance during test sessions based on the simulators feedback, which may put them at an advantage.

To summarize, not all of the investigated advanced cataract surgical modules were able to discriminate between different levels of surgical competency. Only the Iris Expansion Ring extraction – level 2 showed a significant difference between mean score for novices and the more experienced and expert participants for both repetitions of the test (p < 0.001, p = 0.041) and had a significant test–retest reliability (p = 0.012). This could be due to suboptimal software that does not accurately reflect real‐life surgeries. Furthermore, another possible explanation is the composition of the scores for the individual modules. The score for an individual module is made up of different components that measure the participant’s performance within multiple domains such as target achievement, efficiency, instrument handling and tissue treatment. The algorithm from the manufacturer may weigh these different components in such a way that the final score ends up not reflecting actual cataract surgical skill. Another possible explanation for the lack of ability to discriminate between groups may be because the investigated modules sample too few data points, in other words are too short or simplistic (Yudkowsky et al. 2020). Differences in performance will be easier to detect in a detailed test with more data points.

However, it is not unusual to find a lack of validity evidence for simulator metrics. Previous studies investigating validity evidence for other modules on the Eyesi simulator found that only three of six and seven of 13 investigated basic cataract surgical modules showed discriminatory ability, respectively (Selvander & Asman 2013; Thomsen et al. 2015). Another study investigating validity evidence of a training program on the vitreoretinal modules on the simulator found that four of six modules showed discriminatory ability (Vergmann et al. 2017).

Finally, the consequences of the assessment, the consequential validity, refer to the actual consequences of the developed assessment. Can the assessment scores stratified by groups of experience be used to define a pass/fail score (e.g. using Contrasting groups’ method) and explore the consequences of the defined pass/fail score on assessment outcomes (Jorgensen et al. 2018). On the basis of the current study’s results, we did not proceed to define a proficiency criterion, that is defining how much training is enough, as the investigated modules did not possess the needed robustness to complete a consequence analysis. This was because module scores were unable to discriminate between experienced and expert surgeons and in some cases between experienced/experts and novices.

Limitations to our study are mentioned below. Firstly, a significant proportion of the novices included in our study had prior experience with intraocular injections under microscope and wet laboratory simulation that might lead to a reduced difference in performance between groups of participants. However, the inclusion of more experienced trainees makes our findings regarding the advanced modules more relevant. Secondly, the Anterior Vitrectomy module – level 6, did not show any ability to discriminate between novice, experienced and expert surgeons. The module is one of six difficulty levels for anterior vitrectomy and represents one of the most difficult versions of the module. This module was chosen by three expert cataract surgeons (BM, HK and LM), as it corresponded to relevant learning objectives for a cataract surgeon who performed >100 surgeries. However, we can only speculate that this module may have had discriminative ability had another difficulty level been chosen. Secondly, it is relevant to address the wide spread in surgical experience of the participants in our experienced group. Here, the experience of the surgeons ranged from 274 to 1800 surgeries performed, with a median of 1000 surgeries performed. It is clear that it would be less challenging for our test to detect significant differences between the performance scores of distinct groups of participants with narrow ranges of surgical experience within each group and significant differences between groups. However, we wanted to challenge our test and include a group of surgeons often encountered in clinical practice: the experienced surgeon who is no longer novice but not quite an expert yet. This group of surgeons fits the target audience as they are expected to handle their own complications but have not yet transitioned into a supervising role. By nature, this group is more heterogenic than the completely inexperienced novices, or the very experienced experts, and this may have an effect on the results of the study. The effect relates to the prior discussion on the ‘signal/noise ratio’. In our case, the performance difference between the experienced and expert groups, that is ‘the signal’, is too subtle to be detected by our test. This is an insignificant, but nonetheless, important finding, as it would have been considered a substantial strength if this would have been the case.

Consequentially, the investigated modules are not suited for certification of cataract surgeons. However, they may still be relevant in the training of advanced cataract surgical procedures and the handling of complications, such as posterior capsular rupture. Future studies may explore if the experienced cataract surgeon’s performance clinically improves from training using these advanced cataract surgical modules.
